# A trans-diagnostic perspective on obsessive-compulsive disorder

**DOI:** 10.1017/S0033291716002786

**Published:** 2017-03-27

**Authors:** C. M. Gillan, N. A. Fineberg, T. W. Robbins

**Affiliations:** 1 Department of Psychology, New York University, New York, NY, USA; 2 Department of Psychology, University of Cambridge, Cambridge, UK; 3 Behavioural and Clinical Neuroscience Institute, University of Cambridge, Cambridge, UK; 4 National Obsessive Compulsive Disorders Specialist Service, Hertfordshire Partnership NHS University Foundation Trust, UK; 5 Department of Postgraduate Medicine, University of Hertfordshire, Hatfield, UK

**Keywords:** ERN, goal-directed, OCD, RDoC, trans-diagnostic

## Abstract

Progress in understanding the underlying neurobiology of obsessive-compulsive disorder (OCD) has stalled in part because of the considerable problem of heterogeneity within this diagnostic category, and homogeneity across other putatively discrete, diagnostic categories. As psychiatry begins to recognize the shortcomings of a purely symptom-based psychiatric nosology, new data-driven approaches have begun to be utilized with the goal of solving these problems: specifically, identifying trans-diagnostic aspects of clinical phenomenology based on their association with neurobiological processes. In this review, we describe key methodological approaches to understanding OCD from this perspective and highlight the candidate traits that have already been identified as a result of these early endeavours. We discuss how important inferences can be made from pre-existing case-control studies as well as showcasing newer methods that rely on large general population datasets to refine and validate psychiatric phenotypes. As exemplars, we take ‘compulsivity’ and ‘anxiety’, putatively trans-diagnostic symptom dimensions that are linked to well-defined neurobiological mechanisms, goal-directed learning and error-related negativity, respectively. We argue that the identification of biologically valid, more homogeneous, dimensions such as these provides renewed optimism for identifying reliable genetic contributions to OCD and other disorders, improving animal models and critically, provides a path towards a future of more targeted psychiatric treatments.

## Introduction

The mainstay of psychiatric research is the case-control methodology whereby a group of individuals meeting the criteria for a disorder defined by the Diagnostic and Statistical Manual of Mental Disorders, 5th edn (DSM-5; APA, 2013) is compared to a group of healthy controls. Although the DSM has proven extremely useful in establishing a reliable psychiatric nosology that can be applied in a systematic way across centres (Regier *et al.* 2013), and that has led to the widespread development of evidence-based treatments, it has been suggested that shortcomings in the use of these categories for *basic research* are at least partly responsible for the lack of specific and robust associations between psychopathology and underlying neurobiological processes (Hyman, 2007; Sanislow *et al.* 2010).

Obsessive-compulsive disorder (OCD), is a chronic, costly and disabling brain disorder for which existing treatments usually produce disappointing outcomes (Fineberg *et al.* 2014). It is defined in DSM-5 (APA, 2013) by the presence of obsessions and/or compulsions that are time consuming, distressing or disabling. Obsessions are repetitive thoughts, urges or images that are intrusive and unwanted, and that in most individuals cause anxiety or distress, for example recurrent thoughts about accidental death. They are associated with attempts by the individual to suppress or neutralize them with a compulsion. Compulsions are repetitive mental or overt acts that are experienced as being urge-driven either in response to an obsession or according to a rule that must be rigidly applied, and that are aimed at preventing or reducing anxiety or distress or preventing a feared event from happening. However, the compulsion either is not connected in a realistic way with the outcome it is designed to prevent, or is clearly excessive, such as a driver retracing their route to check for signs of an accident after experiencing a minor bump in the road.

DSM-5 acknowledges that these symptoms are not unique to OCD; outlining 14 other disorders (or disorder classes) whose symptoms also broadly fit these criteria, which the clinician must consider in the context of differential diagnosis. These disorders include other members of the obsessive-compulsive and related disorders family, in which obsessions and compulsions are focused either on bodily appearance (body dysmorphic disorder), grooming (trichotillomania and skin picking disorder) or the acquisition of and/or inability to discard personal items (hoarding disorder). Eating disorders are also characterized by behaviours that resemble obsessions and compulsions focused around body size and weight, whereas patients with generalized anxiety disorder (GAD) and depressive disorders experience intrusive, distressing ruminations that are similar to obsessions but are respectively focused on future or past mishaps. In the case of addictive disorders, the urge-driven addictive behaviours become increasingly rigid, stereotyped and compulsive over time, as their function changes from reward-seeking to preventing or neutralizing the distress associated with craving and withdrawal (Everitt *et al.* 2001).

In OCD, the level of insight varies considerably from good to absent both between patients and within the same patient over time. Poor insight has been associated with treatment resistance (Jacob *et al.* 2014). Intrusive, irrational thoughts frequently accompany schizophrenia spectrum and other psychotic disorders, and it can be difficult to distinguish obsessions from delusions, based on phenomenology alone. Moreover, approximately 30% of schizophrenia cases report obsessive-compulsive symptomatology and 14% have been found to have OCD (Swets *et al.* 2014). Some other individuals develop OCD symptoms as a result of environmental factors such as Paediatric Autoimmune Neuropsychiatric Disorder Associated with Streptococcus (PANDAS; Snider & Swedo, 2003).

**Fig. 1.**
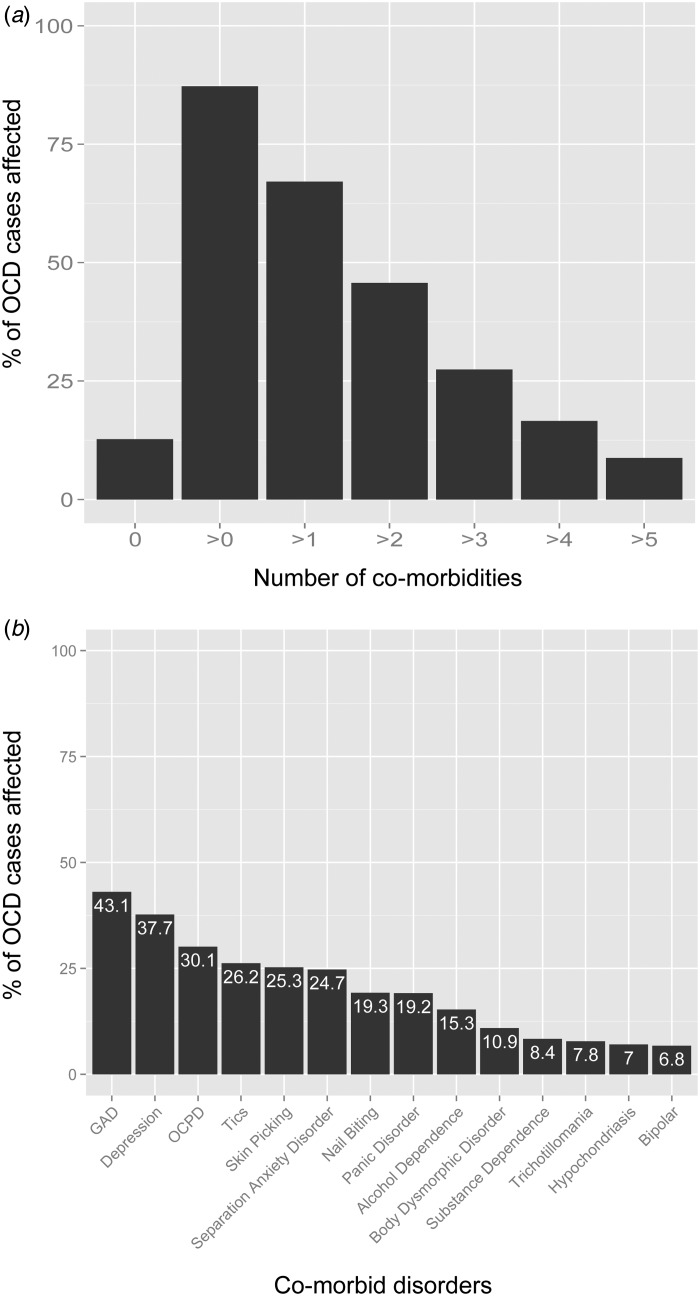
Data from 706 obsessive-compulsive disorder (OCD) patients re-plotted with permission from Nestadt and colleagues (2009). Disorders assessed were obsessive-compulsive personality disorder (OCPD), generalized anxiety disorder (GAD), major depression (recurrent), tics, pathological skin picking, separation anxiety disorder, pathological nail biting, panic disorder, alcohol dependence, body dysmorphic disorder, substance dependence, trichotillomania, hypochondriasis, bipolar disorder. (*a*) Co-morbidity rates in a sample of OCD cases – 87.3% of patients met the criteria for another DSM diagnosis (note: this figure is probably higher as only select diagnoses were assessed). (*b*) The prevalence of specific co-morbid disorders within the OCD cohort.

Even after careful attempts at differential diagnosis of OCD, co-morbidity is present in the majority of cases (Ruscio *et al.* 2010). Other psychiatric disorders, including anxiety, somatoform, impulse control, trauma-related, substance use, and personality disorders, are very common among patients with OCD and can affect adherence and response to treatment (Pallanti *et al.* 2011; Torres *et al.* 2016). Approximately three quarters of individuals with OCD in an epidemiological sample experienced either an anxiety disorder or an affective disorder (Fineberg *et al.* 2013*a*). Most forms of co-morbidity increase distress and impact negatively on family and work relationships, however, disorder-specific effects have also been observed. For example, agoraphobia and GAD were associated with increased OCD severity; bipolar disorder was associated with suicidal acts and panic disorder increased treatment-seeking behaviour (Fineberg *et al.* 2013*a*). In the case of selective serotonin re-uptake inhibitor (SSRI)-resistant OCD, a preferential effect for treatment with dopamine antagonists was seen for those with co-morbid tics (Bloch *et al.* 2006). These findings indicate quite clearly that these and other disorders are not discrete entities, and that the lines between different DSM diagnoses are often blurred (Fig. 1).

The issue of homogeneity across diagnostic categories is coupled with a related problem, *heterogeneity* within diagnostic categories. In the case of OCD, there is a mosaic of diverse phenomenological manifestations (Torres, 2016). A number of authors have attempted to group these phenomenological variables into a smaller number of relatively homogeneous, temporally stable symptom dimensions, using factor analysis of large symptom datasets. This has produced a multidimensional model of OCD that includes symmetry/ordering, hoarding, contamination/cleaning, and obsessions/checking) (Mataix-Cols *et al.* 2005; van den Heuvel *et al.* 2009). The authors propose these symptom dimensions should be understood as a spectrum of potentially overlapping syndromes that may (1) co-exist in any patient, (2) be continuous with normal obsessive-compulsive phenomena, and (3) extend beyond OCD. Although twin studies suggest there might be a genetic influence on dimension-specific risk (van Grootheest *et al.* 2008; Iervolino *et al.* 2011), the neurobiological validity of most of these factors has been difficult to demonstrate, owing to, for example, the lack of cross-study consistency of brain structural abnormalities associated with the various subtypes (Pujol *et al.* 2004; van den Heuvel *et al.* 2009; Alvarenga *et al.* 2012; de Wit *et al.* 2014). The hoarding dimension has proven very useful however, showing a consistently worse response to treatment than other forms of OCD (Mataix-Cols *et al.* 1999; Cullen *et al.* 2007). This particularly impactful finding has provoked a change in DSM-5, such that hoarding symptoms no longer exclusively contribute to a diagnosis of OCD, and are additionally captured by a separate category, ‘hoarding disorder’. Unfortunately, hoarding symptoms do not account for all of the heterogeneity of treatment response in OCD, where for example one meta-analysis determined that the number needed to treat varied from 6–12 for SSRIs compared to placebo (Soomro *et al.* 2008).

The noisy response to treatment that remains to be bridged is likely to be a result of *mechanistic heterogeneity* within the OCD population that we cannot easily identify by analysing symptoms alone. In response, some researchers have called for a shift away from the use of DSM categories, in favour of establishing new and biologically relevant trans-diagnostic traits that may play an important role in multiple disorders, as we currently define them (Robbins *et al.* 2012; Cuthbert & Kozak, 2013). The goal of such an enterprise is to permit evidence-based drug discovery, the development of robust animal models, and eventually realize a future of precision medicine in psychiatry. In a wider context, improved nosology based on more objective tests and criteria would lead to more homogeneous populations of patients for clinical trials, more accurate phenotypes for psychiatric genetics, and the potential for detection of early vulnerability, allowing for early therapeutic interventions that prevent progression to chronic illness.

The National Institute of Mental Health (NIMH) has been an important leader in this regard, launching its Research Domain Criteria (RDoC) initiative (Insel *et al.* 2010), a framework based on the premise that clinical constructs should be defined (and therefore investigated) on the basis of their neurobiological validity. This review will outline such a trans-diagnostic framework for understanding the DSM-5 diagnosis of OCD, outlining putative traits that link genes, molecules, cells, circuits, physiology, behaviour, self-reports and paradigms to clinical phenomenology. The goal is to highlight how a trans-diagnostic approach can shed light on some key puzzles in OCD research, and in so doing showcase the value that can be derived from existing case-control investigations and how this can inform newer attempts at trans-diagnostic psychiatry. Structurally, we will draw heavily on the NIMH's RDoC concepts and criteria, but acknowledge that other new initiatives such as the Roadmap for Mental Health Research (ROAMER) is promoting a similar trans-diagnostic approach (Goschke, 2014). More broadly, we hope to convey the importance of bridging the old with the new. RDoC should be applauded for its focus on biological reality, but perhaps goes too far by ignoring symptom-based approaches entirely. In psychiatry, the symptoms cause the suffering and our focus should remain on understanding and reducing these symptoms, as the patient experiences them.

## Case-control studies point to trans-diagnostic mechanisms in OCD: error-related negativity (ERN) and anxiety

Can OCD be better understood and treated from a dimensional perspective? Evidence for this comes from many sources (Hyman, 2010); of these, perhaps the most compelling (and frustrating) is the lack of specificity of various neurocognitive deficits observed in OCD (Endrass & Ullsperger, 2014; Fineberg *et al.* 2014). For example, ‘response inhibition’, the ability to cancel a prepotent motor response assayed using the stop-signal reaction-time task (Logan, 1994), is reliably impaired in not just OCD, but also attention deficit hyperactivity disorder (ADHD) and schizophrenia (Lipszyc & Schachar, 2010). Pessimistically, one might conclude from this lack of specificity that our current neurocognitive markers are not useful for understanding the aetiology of OCD. Perhaps these markers reflect generalized executive impairment, a consequence of having *any* psychiatric disorder and the burdens thereof. Perhaps these markers are not diagnostic of any specific mental health issue, but simply place individuals into an at-risk state for many. What we will consider in the following sections is an admittedly well-trodden alternative: that it is our psychiatric taxonomy, not our neurocognitive models, that presents a problem for research and that the specificity of any neurocognitive model of a psychiatric diagnosis is fundamentally limited by the extent to which that diagnosis is biologically valid.

How can we determine if a neurocognitive model is specific to a trans-diagnostic phenotype, if not one of the existing DSM disorder categories? What does this mean for interpreting the results of prior work in these DSM diagnosed patient populations? A reasonable starting point is to first identify a model of biological relevance (e.g. neurocognitive or physiological marker) that exhibits *partial* specificity for psychiatric disorders that is perhaps not specific to one disorder, but certainly not common to all. One can then qualitatively identify phenotypic commonalities across the disorders that exhibit mechanistic commonalities and thus formulate hypotheses about trans-diagnostic phenotypes (Robbins *et al.* 2012; Fineberg *et al.* 2014). Publication bias is an obstacle here as studies showing no difference between patient cohorts and controls, which are needed to make a case for specificity, are less likely to be published or even submitted for publication (Rosenthal, 1979). One solution (albeit imperfect) to this problem is studying multiple disorders at once, thereby allowing for direct statistical comparison of disorders that have established deficits in a given domain and disorders that are hypothesized to be no different from controls. Failing this, one can gain insights by leveraging cases where a given neurocognitive mechanism is significantly enhanced in one cluster of disorders, but deficient in another (thereby avoiding publication bias), as is the case for one very promising neurocognitive deficit in OCD that we will discuss in some detail, ERN (Endrass & Ullsperger, 2014).

The ERN is a negative deflection of the event-related potential (ERP) when subjects make an error (Falkenstein *et al.* 1991), listed as the RDoC sub-construct ‘Performance Monitoring’. It is typically measured using electroencephalogram, but functional imaging has been coupled with this technique to confirm that the ERN has its source in the anterior cingulate cortex (ACC) (Debener *et al.* 2005). A host of studies have found enhanced ERN in OCD (Gehring *et al.* 2000; Johannes *et al.* 2001*b*; Ruchsow *et al.* 2005, 2007; Endrass *et al.* 2008, 2010, 2014; Hajcak *et al.* 2008; Stern *et al.* 2010; Riesel *et al.* 2011, 2015; Xiao *et al.* 2011; Hanna *et al.* 2012; Carrasco *et al.* 2013*a*, *b*; Grützmann *et al.* 2014; Klawohn *et al.* 2014; Liu *et al.* 2014) (but see Nieuwenhuis *et al.* 2005; Agam *et al.* 2014; Mathews *et al.* 2015; Weinberg *et al.* 2015), GAD (Ladouceur *et al.* 2006; Weinberg *et al.* 2012, 2015, 2010) (but see Xiao *et al.* 2011), social anxiety disorder (Endrass *et al.* 2014) and depression (Chiu & Deldin, 2007; Holmes & Pizzagalli, 2008, 2010; Georgiadi *et al.* 2011; Aarts *et al.* 2013; Tang *et al.* 2013; Mueller *et al.* 2015) (although less consistently than in generalized anxiety or OCD, see Ruchsow *et al.* 2004, 2006; Olvet *et al.* 2010; Georgiadi *et al.* 2011; Weinberg *et al.* 2012) (Table 1). Accounts regarding the precise function of ERN are varied (Holroyd & Coles, 2002; Yeung *et al.* 2004), but studies have shown that the ERN primes defensive responses, for example by potentiating startle responses (Hajcak & Foti, 2008) and encouraging avoidance behaviour (Frank *et al.* 2005). Together with a qualitative examination of the symptom commonalities across these disorders, this suggests that anxiety might be a reasonable candidate for consideration as a dimensional phenotype related to these deficits. This thesis is supported by work in non-clinical samples, wherein the magnitude of the ERN is associated with self-reported worry (Hajcak *et al.* 2003; Zambrano-Vazquez & Allen, 2014), high levels of trait anxiety (Paulus *et al.* 2004) and OC-symptoms (Hajcak & Simons, 2002; Zambrano-Vazquez & Allen, 2014). The effect appears to be trait (rather than state) dependent; the ERN remains enhanced following successful treatment in both adult and paediatric OCD (Hajcak *et al.* 2008; Riesel *et al.* 2015), does not differ as a result of chronic medication (Stern *et al.* 2010) and an enhanced ERN has been observed in unaffected first-degree relatives of both adult and paediatric OCD sufferers (Riesel *et al.* 2011; Carrasco *et al.* 2013*a*), suggesting that this may be an important endophenotype that predisposes individuals to a range of disorders that involve anxiety.

**Table 1.** Summary of results for studies examining error-related negativity (ERN) across psychiatric disorders

	Unmedicated^a^	ERN^b^	Sample size (case, control)	Task
Obsessive-compulsive disorder				
Gehring *et al.* (2000)		↑	9, 9	Stroop
Johannes *et al.* (2001*b*)	×	↑	10, 10	Cued RT task
Ruchsow *et al.* (2005)		↑	11, 11	Go/NoGo
Nieuwenhuis *et al.* (2005)		n.s.	16, 16	Probabilistic RL
Endrass *et al.* (2008)		↑	20, 20	Flanker
Hajcak *et al.* (2008)^c^		↑	18, 18	Simon
Endrass *et al.* (2010)		↑	22, 22	Flanker
Endrass *et al.* (2010)		n.s.	22, 22	Flanker
Stern *et al.* (2010)^d^		↑	38, 40	Flanker
Xiao *et al.* (2011)^e^		↑	25, 27	Flanker
Riesel *et al.* (2011)		↑	30, 30	Flanker
Hanna *et al.* (2012)^c^		↑	44, 44	Flanker
Carrasco *et al.* (2013*a*)^c^		↑	40, 40	Flanker
Carrasco *et al.* (2013*b*)^c^^,^^e^^,^^f^		↑	26, 27	Flanker
Endrass *et al.* (2014)^e^		↑	24, 24	Flanker
Grützmann *et al.* (2014)		↑	20, 22	Flanker
Agam *et al.* (2014)		n.s.	19, 16	Antisaccade task
Klawohn *et al.* (2014)		↑	26, 26	Flanker
Liu *et al.* (2014)^c^		↑	20, 20	Flanker
Mathews *et al.* (2015)^e^		n.s.	27, 45	Flanker
(Riesel *et al.* (2015)		↑	41, 37	Flanker
Weinberg *et al.* (2015)^e^		n.s.	26, 56	Flanker
Generalized anxiety disorder				
Ladouceur *et al.* (2006)^c^^,^^f^	×	↑	9, 10	Flanker
Weinberg *et al.* (2010)	×	↑	17, 24	Flanker
Xiao *et al.* (2011)^e^		n.s.	27, 27	Flanker
Weinberg *et al.* (2012)^e^	×	↑	26, 36	Flanker
Carrasco *et al.* (2013*b*)^c^^,^^e^^,^^f^		↑	13, 27	Flanker
Weinberg *et al.* (2015)^e^		↑	57, 56	Flanker
Depression				
Ruchsow *et al.* (2004)		n.s.	16, 16	Flanker
Ruchsow *et al.* (2006)		↓	10, 10	Go/NoGo
Chiu & Deldin (2007)		↑	18, 17	Flanker
Holmes & Pizzagalli (2008)	×	↑↑	20, 20	Stroop
Olvet *et al.* (2010)	×	n.s.	22, 22	Flanker
Holmes & Pizzagalli (2010)	×	↑	18, 18	Stroop
Georgiadi *et al.* (2011)^g^		↑	19, 19	Go/NoGo
Georgiadi *et al.* (2011)		n.s.	17, 17	Go/NoGo
Ladouceur *et al.* (2012)^c^	×	↓	24, 14	Flanker
Aarts *et al.* (2013)		↑	20, 20	Go/NoGo
Tang *et al.* (2013)	×	↑	22, 24	Flanker
Mueller *et al.* (2015)	×	↑	14, 15	Probabilistic RL
Weinberg *et al.* (2015)^e^		n.s.	62, 56	Flanker
Other anxiety disorders				
Clemans *et al.* (2012) [PTSD]		↓	10, 10	Flanker
Rabinak *et al.* (2013) [PTSD]	×	n.s.	16, 16	Flanker
Endrass *et al.* (2014) [social]^e^		↑	24, 24	Flanker
Hoarding disorder				
Mathews *et al.* (2015)^e^		↓	14, 45	Flanker
Schizophrenia				
Kopp & Rist (1999)		↓	29, 18	Flanker
Alain *et al.* (2002)		↓	12, 12	Stroop
Bates *et al.* (2002)		↓	21, 21	Go/NoGo
Bates *et al.* (2004)		↓	9, 9	Go/NoGo
Morris *et al.* (2006)		↓	16, 11	Flanker
Morris *et al.* (2008)		↓	26, 27	Probabilistic RL
Mathalon *et al.* (2009)		↓	11, 10	Go/NoGo
Morris *et al.* (2011)		↓	20, 15	Flanker
Foti *et al.* (2012)^e^		↓	33, 33	Flanker
Simmonite *et al.* (2012)^h^		↓	29, 35	Go/NoGo
Horan *et al.* (2012)		↓	16, 14	Flanker
Perez *et al.* (2012)^h^		↓	84, 110	Matching
Houthoofd *et al.* (2013)		↓	12,12	Flanker
Kansal *et al.* (2014)		↓	18,18	Stroop
Minzenberg *et al.* (2014)^e^		↓	73, 54	Stroop
de la Asuncion *et al.* (2015)		↓	22, 21	Simon
Reinhart *et al.* (2015)		↓	19, 18	Probabilistic RL
Bipolar disorder				
Minzenberg *et al.* (2014)^e^		↓	26, 54	Stroop
Kopf *et al.* (2015)		n.s.	20, 18	Flanker
Morsel *et al.* (2014)		↓	16, 14	Flanker
Addiction				
Forman *et al.* (2004) [opiates]		↓	13, 26	Go/NoGo
Chen *et al.* (2013) [opiates]		n.s.	17, 15	Flanker
Franken *et al.* (2007) [cocaine]	×	↓	14, 13	Flanker
Sokhadze *et al.* (2008) [cocaine]		↓	6, 6	Flanker
Schellekens *et al.* (2010) [alcohol]		↑	29, 15	Flanker
Luijten *et al.* (2011) [nicotine]	×	↓	13, 14	Flanker
Zhou *et al.* (2013) [internet]		↓	23, 23	Flanker
Attention deficit hyperactivity disorder				
Liotti *et al.* (2005)^c^^,^^i^	×	↓	10, 10	Stop signal
Wiersema *et al.* (2005)^c^^,^^i^	×	n.s.	22, 15	Go/NoGo
van Meel *et al.* (2007)	×	↓	16, 16	Flanker
Jonkman *et al.* (2007)	×	n.s.	10, 10	Flanker
Burgio-Murphy *et al.* (2007)^c^^,^^e^^,^^i^	×	↑	182, 60	Cued RT task
Burgio-Murphy *et al.* (2007)^c^^,^^e^	×	n.s.	77, 60	Cued RT task
Groen *et al.* (2008)^c^^,^^e^^,^^i^	×	↓	18, 18	Probabilistic RL
Groen *et al.* (2008)^c^^,^^e^^,^^i^		↓	17, 18	Probabilistic RL
Wild-Wall *et al.* (2009)^c^^,^^i^		n.s.	15, 12	Flanker
Zhang *et al.* (2009)^c^		n.s.	14, 14	Go/NoGo
Wiersema *et al.* (2009)		n.s.	23, 19	Go/NoGo
Groom *et al.* (2010)^c^^,^^i^	×	n.s.	23, 19	Go/NoGo
Herrmann *et al.* (2010)^i^	×	↓	34, 34	Flanker
Sokhadze *et al.* (2012)^c^^,^^e^		n.s.	16, 16	Oddball
Groom *et al.* (2013)^c^^,^^i^	×	↓	28, 28	Go/NoGo
Groom *et al.* (2013)^c^^,^^i^		n.s.	28, 28	Go/NoGo
Autism spectrum disorders				
Groen *et al.* (2008)^c^^,^^e^	×	n.s.	18, 18	Probabilistic RL
Vlamings *et al.* (2008)^c^	×	↓	17, 10	Matching
South *et al.* (2010)^h^		↓	24, 21	Flanker
Sokhadze *et al.* (2010)^c^		↓	14, 14	Oddball
Sokhadze *et al.* (2012)^c^^,^^e^		↓	16, 16	Oddball
Henderson *et al.* (2015)^c^		n.s.	38, 36	Flanker

n.s., Non-significant; PTSD, post-traumatic stress disorder; RT, reaction time; RL, reinforcement learning.

a Unmedicated were free of selective serotonin re-uptake inhibitors or antipsychotics for at least 2 weeks, and free of stimulants for 17 h. In the case of addiction, unmedicated applies when subjects meet the above criteria and are abstinent.

b Studies were included if they reported results of ERN analysis (either ERN or ERN corrected for correct-related negativity), included a case-control comparison, and reported generic ERN effects using a task that could be compared across studies.

c Paediatric/adolescent sample.

d Compared medicated and unmedicated groups, and no medication effect was observed.

e Compared to other diagnoses in trans-diagnostic design.

f Mixed anxiety (mostly generalized anxiety disorder).

g Remitted patients.

h Mixture adult and adolescent.

i Attention deficit hyperactivity disorder combined-type only.

Critically, the postulate that an enhanced ERN is a useful marker of an anxious phenotype rests on the *specificity* of this effect. We can begin to, albeit imperfectly, approach this issue using existing work in diagnosed patients – specifically if enhanced ERN is observed in disorders not in part characterized by pathological anxiety, then this suggests there is more evidence for the pessimistic view suggested earlier. Fortunately, evidence for such specificity is abundant for this particular neurocognitive marker; a large body of work has showed that a significantly *decreased* ERN magnitude is characteristic of a putatively distinct cluster (or clusters) of disorders, most consistently schizophrenia (Kopp & Rist, 1999; Alain *et al.* 2002; Bates *et al.* 2002, 2004; Morris *et al.* 2006, 2008, 2011; Mathalon *et al.* 2009; Foti *et al.* 2012; Horan *et al.* 2012; Perez *et al.* 2012; Simmonite *et al.* 2012; Houthoofd *et al.* 2013; Kansal *et al.* 2014; Minzenberg *et al.* 2014; de la Asuncion *et al.* 2015; Reinhart *et al.* 2015), but also bipolar disorder (Minzenberg *et al.* 2014; Morsel *et al.* 2014) (but see Kopf *et al.* 2015), autism spectrum disorders (Vlamings *et al.* 2008; Sokhadze *et al.* 2010, 2012; South *et al.* 2010) (but see Groen *et al.* 2008; Henderson *et al.* 2015) and various forms of addiction (Forman *et al.* 2004; Franken *et al.* 2007; Sokhadze *et al.* 2008; Luijten *et al.* 2011; Zhou *et al.* 2013) (but see Schellekens *et al.* 2010; Chen *et al.* 2013). Moreover, the amplitude of the ERN was found to predict treatment adherence for substance abuse (Steele *et al.* 2014) and decreases in the ERN are associated with self-reported impulsivity (Potts *et al.* 2006). Results in ADHD are mixed with some studies showing decreased ERN (Liotti *et al.* 2005; van Meel *et al.* 2007; Groen *et al.* 2008; Herrmann *et al.* 2010; Groom *et al.* 2013), but most reporting no differences (Wiersema *et al.* 2005, 2009; Groen *et al.* 2008; Wild-Wall *et al.* 2009; Zhang *et al.* 2009; Groom *et al.* 2010; Sokhadze *et al.* 2012) and only one study reporting an increase (Burgio-Murphy *et al.* 2007) (Table 1). Together, these data provide convergent evidence that an *enhancement* of the ERN is not ubiquitous in psychiatry, but rather appears to be directionally specific to disorders characterized predominantly by anxiety. This therefore may constitute a trans-diagnostic marker relevant for many disorders involving anxiety, including OCD.

## Validating a trans-diagnostic mechanism: goal-directed learning and compulsivity

The suggestion that the consistent finding of enhanced ERN in OCD in fact reflects a trans-diagnostic phenotype representing anxiety is perhaps a compelling one given the pattern of results described above. However, without direct comparison across disorders and/or discrete symptom dimensions, conclusions regarding its phenomenological specificity cannot be drawn (indeed ‘anxiety’ is intended to serve as a placeholder, until such work is complete). We will now highlight an approach that can take this next step, drawing on data from an independent line of research in OCD. This body of research centres on the theory that an imbalance between ‘goal-directed control’ and ‘habit-learning’ drives symptoms in OCD (see Gillan & Robbins, 2014 for detailed account) (see Supplementary material), such that compulsive behaviours in OCD are not goal-directed responses to anxiety/perceived threat, but are in fact stimulus-evoked, goal-insensitive habits (Dickinson, 1985). This model suggests that compulsions are not a search for safety; they are a result of the need to realize a link that has developed between a stimulus or context and a given set of responses. In this view, the subjective experiences that accompany habits, such as ‘not just right experience’ (Coles *et al.* 2005; Ecker & Gönner, 2008), or more complex obsessive thoughts are the result of the compulsive urge, not the cause. For example, there is preliminary evidence to suggest that obsessive thoughts can even arise as a result of compulsive habit formation in OCD, and not the other way around (Gillan *et al.* 2014*b*; Gillan & Sahakian, 2015), constituting a sharp divergence from most cognitive models of OCD that focus on obsessive thoughts (Salkovskis, 1985). The original habit hypothesis of OCD was based on a convergence of neurobiological data illustrating that the pathophysiology of OCD overlaps extensively with that supporting the balance between goal-directed behaviour and habits (Graybiel & Rauch, 2000; Dolan & Dayan, 2013). Empirical studies have found broad support for this idea; OCD patients show a reliable tendency to form habits excessively in both appetitive and aversive learning contexts (Gillan *et al.* 2011, 2014*b*). Based on convergent evidence from follow-up investigations utilizing neuroimaging and computational modelling (Gillan *et al.* 2014*a*, 2015*a*; Voon *et al.* 2014), the working model theorizes that deficits in goal-directed control, mediated by the caudate and medial orbitofrontal cortex, are responsible for the excessive habits observed in OCD in the laboratory (Gillan & Robbins, 2014). Clinically, these habits might manifest as compulsive behaviours and/or higher-order habitual needs or goals (Cushman & Morris, 2015).

Addressing the trans-diagnostic potential of this neurocognitive mechanism, goal-directed control has been suggested to play a role in a range of disorders that are clinically characterized by repetitive behaviours that persist despite negative events, or are experienced as ‘out of control’ (Gillan *et al.* 2016*a*). These disorders include addiction (Everitt & Robbins, 2005), eating disorders (Godier & Park, 2014) and Tourette's syndrome (Groenewegen *et al.* 2003), and although this area of research is still in its infancy, the first empirical investigations have found evidence for goal-directed deficits in these disorders (Sjoerds *et al.* 2013; Voon *et al.* 2014; Delorme *et al.* 2015; Ersche *et al.* 2016) (Table 2). As such, the candidacy of goal-directed deficits as a trans-diagnostic marker has been acknowledged in the RDoC matrix, which flags several relevant theoretical domains, i.e. ‘Habit’, ‘Goal Selection, Updating, Representation and Maintenance’ and ‘Response Selection, Inhibition or Suppression’. However, studies have recently been published showing similar deficits in goal-directed control in schizophrenia (Morris *et al.* 2015), autism spectrum disorder (Alvares *et al.* 2016), and most problematically, social anxiety disorder (Alvares *et al.* 2014, 2016), which is not considered clinically to be a ‘compulsive’ disorder (Table 2).

**Table 2.** Summary of results for studies examining goal-directed learning

	Unmedicated^a^	Goal-directed control	Sample size (case, control)	Task
Obsessive compulsive disorder				
Gillan *et al.* (2011)		↓	20, 20	Devaluation
Gillan *et al.* (2014*b*)		↓	25, 25	Devaluation
Gillan *et al.* (2014*a*)		↓	20, 20	A-O learning
Voon *et al.* (2014)^b^	×	↓	32, 96	MB learning
Gillan *et al.* (2015*a*)^c^		↓	37, 33	Devaluation
Social anxiety disorder				
Alvares *et al.* (2014)		↓	23, 23	Devaluation
Alvares *et al.* (2016)^b^		↓	20, 19	Devaluation
Schizophrenia				
Morris *et al.* (2015)		↓	18, 18	Devaluation
Addiction				
Sjoerds *et al.* (2013) [alcohol]		↓	31, 19	A-O learning
Voon *et al.* (2014) [meth]^b^		↓	22, 66	MB learning
Voon *et al.* (2014) [alcohol]^b^	×	n.s.	30, 90	MB learning
Ersche *et al.* (2016) [cocaine]		↓	72, 53	Devaluation
Tourette syndrome				
Delorme *et al.* (2016)	×	↓	17, 17	Devaluation
Delorme *et al.* (2016)		n.s.	17, 17	
Autism spectrum disorders				
Geurts & de Wit (2013)^d^		n.s.	24, 24	Devaluation
Alvares *et al.* (2016)^b^		↓	17, 19	Devaluation
Eating disorders				
Voon *et al.* (2014) [binge eating]^b^	×	↓	31, 93	MB learning

n.s., Non-significant.

Goal-directed learning was measured using devaluation, model-based (MB) learning or action-outcome (A-O) learning test. The latter two measures are proxies for devaluation sensitivity (Gillan *et al.* 2011, 2015*b*).

a Unmedicated were free of selective serotonin re-uptake inhibitors or antipsychotics for at least 2 weeks, and free of stimulants for 17 h. In the case of addiction, unmedicated applies when subjects meet the above criteria and are abstinent.

b Compared multiple diagnostic groups.

c Compared medicated and unmedicated groups, and no medication effect was observed.

d Paediatric sample.

Using a case-control design, it is difficult to disentangle a genuine lack of mechanistic specificity, as these data might indicate, from ‘DSM measurement error’, the confounding influence of co-morbid psychiatric disorders. For example, in the aforementioned studies in social anxiety disorder patients (Alvares *et al.* 2014, 2016), because patients presented with multiple co-morbid disorders, one cannot know if the results are attributable to social anxiety symptoms or one or more of their co-morbid conditions. Indeed, like most disorders, OCD rates are higher in the social anxiety population (Grant *et al.* 2005). To get around this issue, some researchers (ourselves included) have endeavoured to recruit only ‘pure cases’ of OCD, i.e. those that do not meet the criteria for any other psychiatric diagnoses (Table 2). Although somewhat effective, the problem with this approach is that it assumes that being one criterion short of diagnosis of depression, for example, is the same as having no depressive symptoms at all. In reality, OCD patients recruited in this way consistently have higher levels of sub-threshold symptoms of multiple other disorders, e.g. depression and anxiety (Gillan *et al.* 2011). While these confounding variables could be controlled for statistically, most patient studies simply do not have the necessary power to do so. Indeed, the difficulty in recruiting sufficient sample sizes for research studies with patients is compounded by the increasing number of (possibly confounding) disorders defined by the DSM in every new edition.

**Fig. 2.**
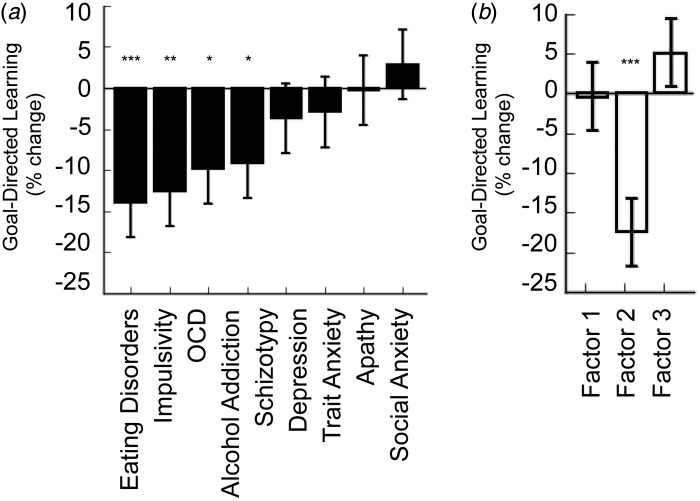
Validating a trans-diagnostic dimension. (*a*) The strength of the association between self-report symptoms of various DSM disorders and deficits in goal-directed control. The pattern is strikingly non-specific. (*b*) The association between goal-directed deficits and three ‘trans-diagnostic symptom dimensions identified in a data-driven factor analysis. Factor 1 corresponds to ‘Anxious Depression’, Factor 2 is ‘Compulsive Behavior and Intrusive Thought’, and Factor 3 is ‘Social Withdrawal’. The association between Factor 2 (‘Compulsive Behavior and Intrusive Thought’) and deficits in goal-directed learning is greater than that of any of the nine DSM-inspired questionnaires and crucially, the relationship exhibits excellent specificity with respect to ‘non-compulsive’ aspects of psychopathology, i.e. Factor 1 (‘Anxious Depression’) and Factor 3 (‘Social Withdrawal’). Data reproduced with permission from Gillan *et al.* (2016*b*). *p < 0.05, **p < 0.01, ***p < 0.001.

A novel solution to this problem was presented in a recent study that eschewed the traditional case-control design in favour of the greater sample sizes (and in turn statistical power) that can be achieved by leveraging normal variability in a large general population sample (Gillan *et al.* 2016*b*). The study tested if the relationship between goal-directed control and psychopathology constitutes a trans-diagnostic trait by testing its generalizability and specificity. Just under 2000 subjects were recruited online and completed self-report questionnaires assessing multiple psychiatric symptoms and completed a web-based task that assessed goal-directed performance (i.e. ‘model-based learning’; Daw *et al.* 2011). In line with the preceding case-control literature, normal variation in self-reported OCD symptomatology (Foa *et al.* 2002) was associated with decreases in goal-directed control in two separate samples collected in this study (also see a recent independent replication: Snorrason *et al.* 2016). Importantly, this effect was generalizable to symptoms of other aspects of compulsive psychopathology (as previously characterized in the literature: Everitt & Robbins, 2005; Hogarth *et al.* 2012; Godier & Park, 2014). Specifically, eating disorders (Garner *et al.* 1982), impulsivity (Patton *et al.* 1995) and alcohol addiction (Saunders *et al.* 1993) were also associated with deficits in goal-directed control, suggestive of a trans-diagnostic deficit linking these disorders. Crucially, these deficits were not associated with symptoms of trait anxiety (Spielberger *et al.* 1983), depression (Zung, 1965), social anxiety (Liebowitz, 1987) or apathy (Marin *et al.* 1991). Schizotypy (Mason *et al.* 2005) sat somewhere in the middle, showing a marginal association with goal-directed deficits (Fig. 2*a*).

Although these results are suggestive of generalizability and specificity, this kind of analysis does not take us much further than work in diagnosed patients, save to say that these effects appear to be continuous in the general population rather than being specific to diagnosed groups. A more important question is what the psychiatric phenotype that explains this pattern of results? Is there a trans-diagnostic symptom dimension shared by some disorders (e.g. OCD, addiction, eating disorders), but not others (e.g. social anxiety, depression)? To answer this question empirically, this study employed a two-stage validation methodology. First, a factor analysis was carried out on psychiatric self-report data to identify a smaller set of clinical phenotypes that more parsimoniously explain the self-report symptom data. This approach revealed that three factors could replace the nine original questionnaires and of particular interest was a factor linking disorders at the level of compulsive behaviour and thought, so-called ‘Compulsive Behaviour and Intrusive Thought’. This factor comprised select features of OCD, eating disorders, addiction and some aspects of impulsivity and schizotypy that pertain to a loss of control over repetitive behaviour and thought. Next, this phenotype was tested for biological validity by assessing if subjects’ scores on this factor predicted goal-directed performance in an independent analysis. ‘Compulsive Behaviour and Intrusive Thought’ was found to be more predictive of goal-directed deficits than any DSM-inspired questionnaire collected in this study (Fig. 2*b*), illustrating the power of data-driven clinical phenotyping. For example, this trans-diagnostic symptom dimension predicted task performance better than total scores on questionnaires measuring severity of DSM disorders such as OCD, eating disorders and addiction. Crucially, this relationship was dissociable from other trans-diagnostic phenotypes identified in this study, ‘Anxious-Depression’ and ‘Social Withdrawal’ (which showed no relation to goal-directed deficits), which could be compared directly in this study, providing compelling evidence for the specificity of this deficit to ‘Compulsive Behaviour and Intrusive Thought’. This approach showcases how in an appropriately powered general population sample, a neurocognitive model can be tested for both generalizability and specificity, leading to a data-driven definition of a neurobiologically validated clinical phenotype.

## Going deeper: ‘units of analysis’

**Fig. 3.**
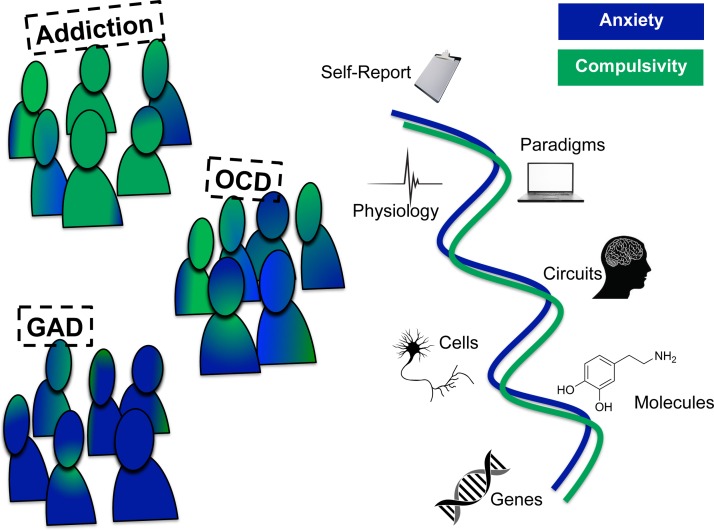
A trans-diagnostic approach to obsessive-compulsive disorder (OCD). The predominant symptoms of OCD may result from complex interaction between independent trans-diagnostic dimensions, of which anxiety and compulsivity are good (but not the only) candidates. In this simplified schematic, we show just two putative trans-diagnostic psychiatric dimensions, there are of course many others. The task for basic research, outlined in the Research Domain Criteria initiative is to understand these dimensions at units of analysis, linking self-report symptoms (such as anxiety and compulsivity) to paradigms, physiology, circuits, cells, molecules and genes. GAD, Generalized anxiety disorder.

In tandem with new approaches to empirically define psychiatric phenotypes, the task for basic research is to delineate the neurobiology that underpins them. RDoC proposes that this should be done, where appropriate, using *units of analysis* that link clinical phenomenology (e.g. self-reports) to behaviour/physiology (via paradigms) to the underlying circuit, cells, molecules and finally, genes. The aims of this approach are to facilitate evidence-based drug-development (e.g. based on pharmacological underpinnings), to guide new therapeutic approaches such as psychological therapies (e.g. based on neurocognitive changes) or brain stimulation (e.g. based on knowledge of brain circuits), and to identify and treat those at risk (e.g. using genetic tests) (Fig. 3).

Here, we will highlight just a couple of examples drawing on units of analysis that have the most direct implications for treatment. Might the ERN be useful for deciding on which treatment to prescribe to which individual? To begin to answer this, we must review what is known about the pharmacology of the ERN (i.e. as a molecular unit of analysis). Benzodiazepines, specifically alprazolam (Riba *et al.* 2005), lorazepam (de Bruijn *et al.* 2004) and oxazepam (Johannes *et al.* 2001*a*), reduce the amplitude of the ERN. This suggests a potential role for GABA (which is thought to directly inhibit ACC functioning) in the ERN and with that, a putative biological basis for the therapeutic effect of these drugs for GAD and therefore plausibly also for anxiety as a trans-diagnostic trait (Ravindran & Stein, 2010). Unfortunately, this theory runs into issues when one considers that benzodiazepines are ineffective for treating OCD (e.g. clonazepam, Hollander *et al.* 2003). Moreover, antidepressants, such as paroxetine and mirtazapine, which appear to have no effect on the ERN (de Bruijn *et al.* 2004, 2006), have good efficacy for treating both OCD and GAD. This apparent lack of pharmacological specificity is compounded by research on dopamine, which is probably been the most studied neurotransmitter with respect to the ERN. Studies in this area have shown that amphetamine administration increases the amplitude of the ERN (de Bruijn *et al.* 2004), while antipsychotics such as haloperidol and olanzapine reduce it (Zirnheld *et al.* 2004; de Bruijn *et al.* 2006). Although antipsychotics can be useful for OCD in particularly treatment resistant cases, GAD does not respond to antipsychotics (Ravindran & Stein, 2010).

Thus, the question of how an ERN measurement can help inform treatment response across disorders is far from clear. It may be the case that combined measures, such as ERN, structural scans and self-report, are needed to produce a useful therapeutic signal. For example, although the core symptoms of OCD do not typically reduce following benzodiazepines, this drug class might be useful for decreasing anxiety in certain OCD patients – and these particularly anxious patients might be driving the ERN findings from studies examining this patient population at the group level. Alternatively, the ERN might have no specific causal role for psychiatry whatsoever, it might *arise* from a range of discrete upsets to neural harmony – longitudinal designs are needed to answer these questions definitively.

Turning to circuits, cells and the link between self-report compulsivity and deficits in goal-directed control (Gillan *et al.* 2016*b*), studies point to the involvement of the medial orbitofrontal cortex (mOFC) and caudate based on their involvement in goal-directed control across species (Dolan & Dayan, 2013) and convergent data suggesting these are critically involved in OCD and other compulsive disorders. For example, hyperactivity in the caudate nucleus is linked to failures in goal-directed behaviour observed in OCD patients (Gillan *et al.* 2015*a*). In terms of brain structure, deficits in goal-directed control are associated with reductions of grey-matter volume in the caudate and mOFC in healthy individuals (Voon *et al.* 2014). Moreover, in this same study, the observation that these regional volumes are reduced in binge-eating disorder patients was statistically explained by their deficits of goal-directed control relative to controls (Voon *et al.* 2014). Although imaging studies in humans are important for validating animal models of psychiatric populations, animal models are crucial as they can uniquely assess causality (Ahmari, 2015). To this end, one exemplary study showed that chronic stimulation of mOFC neurons that project to the ventromedial striatum [i.e. mimicking the hyperactivity observed in human OCD patients (Gillan *et al.* 2015*a*)] induces compulsive grooming behaviour in mice (Ahmari *et al.* 2013). Furthermore, the authors found that excessive grooming was ameliorated with chronic SSRI treatment, the first line pharmacotherapy for OCD (Fineberg *et al.* 2013*b*), thereby showing convergence across multiple units of analysis, from behaviour to brain circuits to molecules. What remains to be seen is whether this kind of chronic stimulation also induces deficits in goal-directed control in tandem with compulsive grooming, and if deficits in goal-directed control are remediated by treatment with SSRIs. These are testable hypotheses that, if supported, could mean that computerized tasks that assess this neurocognitive mechanism might be used to inform treatment allocation in the future. In addition to guiding pharmacotherapy decisions, work at the circuit level can be used to select target sites for deep-brain stimulation, of which there are many candidates for OCD (Mallet *et al.* 2008; Nuttin *et al.* 2008; Denys *et al.* 2010; Greenberg *et al.* 2010) and indeed to guide the allocation of distinct forms of behavioural therapy (Saxena *et al.* 2009). The question of whether deficits in goal-directed control diminish with successful treatment (and are akin to state phenomena) or remain elevated and stable (trait) awaits investigation. But even more interesting in our minds is the question of whether or not goal-directed performance at baseline predicts better response to one treatment over another. This may be how the success of the marker should be ultimately judged.

## Precision therapeutics: insights and trans-diagnostic opportunities

Although defining biologically valid clinical phenotypes as accurately and discretely as possible is valuable, it may not be the most important thing that trans-diagnostic psychiatry research can contribute. Even if a phenotype such as ‘compulsivity’ possesses good neurobiological validity, specificity and homogeneity, this may not translate into homogeneity of treatment response. Rather, discrete neural pathophysiology may give rise to common network-level dysfunctions and behavioural manifestations, and these distal phenotypic markers might therefore offer minimal insight for clinicians in deciding on whether or not an SSRI will be effective in an individual. One way to address this is to work from the bottom-up – to take a step back from phenotypic classification and focus instead on establishing robust and direct links between neurocognitive markers and treatment response. This kind of work has already begun, typically within the confines of one diagnostic category or another and we will briefly mention some of the most promising data. We will take a particular focus on pharmacological interventions, but the general approach can similarly be applied to behavioural interventions. Then we will highlight how treatment prediction research involving sufferers of many DSM categories that are prescribed the same treatment is likely to be an important approach of the future.

Taking neural markers as an example, several studies have had early success at using functional imaging at the group level to predict treatment response within the OCD population (for review see Ball *et al.* 2014). One potential marker of SRI response in OCD is reduced baseline activity in the OFC and ACC (Swedo *et al.* 1992, 1989; Brody *et al.* 1998; Saxena *et al.* 1999). Unfortunately several studies that came later failed to replicate (Saxena *et al.* 2003; Carey *et al.* 2004; Ho Pian *et al.* 2005; van der Wee *et al.* 2007), finding no significant effects or in some cases entirely different neural markers. A recent study tested whether a task-related ACC measure like ERN might fare better than these resting studies at predicting clinical response. Although the result should be taken cautiously as the sample size was particularly small (only 10 patients returned at post-treatment assessment), the authors were unable to find a relationship between baseline ERN and cognitive behaviour therapy response in a paediatric OCD sample (Hajcak *et al.* 2008).

One possibility is that the predictive value of ACC activity (if there is any) could be exclusive to a putative anxiety dimension and this might get washed out in studies relying on heterogeneous diagnostic categories and small samples. Recruiting large samples across diagnostic categories and recording changes in the symptoms of dissociable psychiatric dimensions like anxiety and compulsivity might help isolate reliable effects. Indeed, in the one study that adopted a trans-diagnostic approach, recruiting individuals with OCD, depression or both, the authors were able to identify *dissociable* neural markers of subsequent depressive *v*. obsessive-compulsive response to paroxetine (Saxena *et al*. 2003). This means that not only is the same drug effective in treating distinct psychiatric problems, its efficacy at treating depression and OCD symptoms (sometimes in the same individual) is predicted by dissociable patterns of abnormal baseline neural activity. Given that individuals with a diagnosis of OCD are aetiologically and phenotypically heterogeneous, the task for future research is not to identify mean baseline activity patterns in responders *v*. non-responders on some singular measure of function, but to use large datasets to tease apart the mechanisms through which a given drug can reduce symptoms of largely independent psychiatric dimensions, and use these insights to identify the patients for whom a symptom reduction is likely to happen following treatment.

To conclude this section with some practical statements, morphometric predictors of SRI response in OCD already appear to be somewhat more robust than functional ones. The only two structural MRI studies to date have both shown that decreased grey matter in the lateral OFC is also linked to SRI response (Hoexter *et al.* 2013, 2015). Hoexter and colleagues showed that OFC grey-matter volume had 77% sensitivity and 81% specificity at predicting SRI response in OCD. If these figures hold up to replication using the same analysis pipeline, the interpretation is that if this marker were used to determine whether or not an SRI is prescribed to a drug-naive individual, just two out of every 10 people assigned to SSRI would not respond, while another two of the 10 not assigned the treatment would have missed out on the benefits of the SSRI (Hoexter *et al.* 2015). This would make it a useful tool for clinicians, were MRI scans not expensive and impractical for most clinics to use diagnostically (at least for now). This is where we suggest cognitive neuroscience might be able to intervene by replacing brain scans with cognitive tasks that are good indicators of neurobiology, such those linked to normal and pathological variation in OFC grey-matter volume, of which goal-directed control is one notable example (Voon *et al.* 2014). Cognitive behavioural therapy for OCD similarly might benefit from the addition of components that focus more directly on habit formation and goal-directed control in those patients for whom it is more relevant. For example, one might focus psychoeducation: patients could be taught about identifying habit ‘trigger’ situations, or identifying the development of new habits that might later develop into more elaborate compulsions. Alternatively, habit-reversal training (Snorrason *et al.* 2015) may be used to complement standard exposure and response prevention techniques. These behavioural treatments might be further enhanced with targeted pharmacotherapy, for which basic data are accruing (Lovinger, 2010).

## Combining trans-diagnostic with existing approaches

We have argued strongly in favour of the trans-diagnostic model as a rational approach to advance basic science research in psychiatry, but must make clear that to date this approach has yet to facilitate gene discovery or the development of new, better treatments. It will take time for sufficient data to accrue and as such, dismissing symptom-based approaches entirely is premature. Existing clinical trial datasets, based on patients grouped according to conventional diagnostic criteria such as DSM, have demonstrated a clear specificity of effect for certain psychiatric treatments [e.g. serotonin *v*. noradrenaline reuptake inhibitors in OCD (Fineberg *et al.* 2014)], and a magnitude of efficacy for some of our treatments commensurate with the effect sizes seen in other branches of medicine (Davis *et al.* 2011; Leucht *et al.* 2015). We have also seen that specific drug response can be linked to easily observable symptoms, such as the presence of tics (Bloch *et al.* 2006). The hope is that by revisiting how we define disorder and/or dimensions using biological and cognitive data will further enhance the precision with which treatments can be delivered to individuals. In addition, new ways of assessing the outcomes of treatment trials, e.g. using statistical techniques such as group-based mixture models, that divide ‘diagnostic’ patient samples into clinically meaningful subgroups with greater predictive value, hold promise for the advancement of personalized psychiatric treatment (Thase *et al.* 2011). Thus, most progress is likely to be made with a balanced approach, taking into account and if possible combining the most valid and reliable diagnostic and trans-diagnostic methodologies.

## Conclusion

This paper presented a trans-diagnostic perspective on OCD – a diagnostic category – that selectively focused on just two putative mechanisms relevant for our understanding of anxiety and compulsivity in OCD. These candidate mechanisms illustrate how two orthogonal trans-diagnostic mechanisms might converge to produce a phenotype that is currently defined as a unitary category. Specifically, avoidance compulsions, obsessions and anxiety are the defining clinical features of OCD according to DSM-5 (APA, 2013). As the symptom complex that this triad produces is unlikely to be underpinned by one specific cognitive/neural mechanism, breaking OCD down into orthogonal trans-diagnostic mechanisms may be of benefit for developing translational models – particularly for animal models that are crucial for causal mechanistic investigations. This research initiative has just begun for OCD, and many candidate dimensions await empirical validation or rejection. This can be achieved in many different ways, just a few of which have been outlined here (see other good examples: Fair *et al.* 2012; Brodersen *et al.* 2014), and at many units of analysis. Most importantly, psychiatric diagnostic tools of the future must focus on maximizing predictive value, refining treatment allocation (both behavioural and pharmacological), identifying prospects and tools for early intervention, determining risk and clinical course. To do this, we need a new wave of focused longitudinal studies in psychiatry that can assess which cognitive tests or neural markers possess the most clinically useful properties. While studies of this kind have been historically difficult to conduct, costly and complicated, the field is already rising to the challenge by developing new methodologies (Brodersen *et al.* 2014; Gillan & Daw, 2016) that will help us to put biological psychiatry to the clinical test.

## Supplementary material

For supplementary material accompanying this paper visit http://dx.doi.org/10.1017/S0033291716002786. click here to view supplementary material
