# Supplementary material for: A trans-diagnostic perspective on obsessive-compulsive disorder
Source: Psychol Med. 2017 Mar 27;47(9):1528–48. doi: 10.1017/S0033291716002786 (PMC5964477; doi:10.1017/S0033291716002786)
Supplement: Supplementary file 1 [file S0033291716002786sup001.docx]

**SUPPLEMENTARY MATERIAL**

**The Habit Hypothesis of OCD**

Goal-directed control reflects animals’ ability to make flexible choices, based on what they want at a given moment, and knowledge of what action is most likely to get it (Dickinson, 1985). This kind of prospective, model-based decision-making sits in contrast to habits, which are behaviors that we repeat when faced with familiar environments, because those behaviors were previously rewarded in those settings (Graybiel, 1998). Both modes of action selection are important for optimal behavior, representing something akin to a classic speed-accuracy trade-off. Habits do not require much effort; they can be executed while we are otherwise taxed, either cognitively (Otto *et al.* 2013) or by external stressors (Schwabe & Wolf, 2009). Moreover, by definition, habits are *usually* appropriate to the situation at hand, and therefore constitute a reliable way to navigate our daily routines. Goal-directed behavior takes more cognitive effort (Otto *et al.* 2013), but this makes behavior more precise and finely tuned to the requirements of the moment, e.g. when our motivations change (Adams, 1982).

Several studies have identified a consistent impairment in goal-directed control in OCD patients relative to healthy controls (Gillan *et al.* 2011, 2014*a*,*b*, 2015*a*, Voon *et al.* 2014). These failures in goal-directed control are believed to cause patients to get stuck in repetitive habits (Gillan *et al.* 2015*b*). Unlike existing cognitive models, this habit hypothesis of OCD is indifferent to the specific content of compulsive behaviors (e.g. washing versus checking, relating to NJREs or HA). Instead it accounts for the key feature that all compulsions share – they persist despite no longer producing a valuable outcome, and in most cases despite clear negative consequences. Support for this tenet comes from the finding that goal-directed deficits observed in OCD patients in the laboratory are profoundly domain-general – they are observable despite bearing no relation to existing symptoms, and regardless of whether actions are designed to avert negative events or to gain monetary rewards. These domain-generality is further supported by the recent extension of this model to ‘compulsivity’, which captures obsessions and compulsion relating to weight or shape and drug addiction (Voon *et al.* 2014; Gillan *et al.* 2016*b*).

In agreement with cognitive theories of OCD, laboratory studies have found that in OCD habits are not experienced as simple motor repetitions, devoid of conscious representation. Rather, they are associated with a premonitory urge. One possibility is that obsessions in OCD arise following a transition from an initial urge to realize a habit (similar to a NJRE) to a much more elaborate *post-hoc* cognitive explanation for repetitive behavior that develops with time and repetition (Gillan & Sahakian, 2015). However, there is certainly room for both accounts. In OCD, intrusive thoughts [which we are prevalent outside the OCD population (Rachman and de Silva, 1978)] might in some cases act as seeds around which elevated habit-forming tendencies become compulsive.
